# Editorial: Molecular mechanisms in testicular germ cell tumors

**DOI:** 10.3389/fonc.2024.1427834

**Published:** 2024-05-17

**Authors:** João Lobo, Andres M. Acosta, Michal Mego

**Affiliations:** ^1^ Department of Pathology, Portuguese Oncology Institute of Porto/Porto Comprehensive Cancer Centre (Porto.CCC), Porto, Portugal; ^2^ Cancer Biology and Epigenetics Group, Research Center of IPO Porto (CI-IPOP)/RISE@CI-IPOP (Health Research Network), Portuguese Oncology Institute of Porto (IPO Porto)/Porto Comprehensive Cancer Centre (Porto.CCC), Porto, Portugal; ^3^ Department of Pathology and Molecular Immunology, School of Medicine & Biomedical Sciences, University of Porto (ICBAS-UP), Porto, Portugal; ^4^ Department of Pathology Indiana University, Indianapolis, IN, United States; ^5^ 2nd Department of Oncology, Faculty of Medicine, Comenius University, Bratislava and National Cancer Institute, Bratislava, Slovakia

**Keywords:** biomarkers, testicular cancer, molecular pathology, germ cell tumors, targeted therapy

Testicular germ cell tumors (TGCTs) are the most common solid cancers of young-adult men, and their incidence is increasing due to still poorly understood environmental factors exerting effect *in utero* and postnatally. TGCTs are the most common neoplasms of the testis, but may also occur in the ovaries and in extragonadal sites along the midline of the body (reflecting the migration of primordial germ cells during development), such as the mediastinum or the retroperitoneum (1).

Germ cell tumors (GCTs) are often called “developmental cancers” since the different types reflect the developmental potential of their cell of origin, including their epigenetic background (2). The better understanding of developmental biology led to advances in recognition of clinically relevant biomarkers, including classical serum tumor markers human chorionic gonadotropin (HCG) and alpha-fetoprotein (AFP), to pluripotency factors (such as OCT3/4), and embryonic microRNAs of the 371~373 cluster (3).

While most TGCT patients present with limited disease and are treated with orchiectomy alone, some benefit from adjuvant treatment. The molecular background of TGCTs makes them particularly sensitive to platin-based chemotherapy, being one of the most curable solid cancers. Their sensitivity to chemotherapy can be explained in part by the presence of globally demethylated genomes and active pro-apoptotic pathways (e.g., p53), among other mechanisms (4). However, some patients develop relapses, and a proportion of those exhibit resistance to chemotherapy, with poor clinical outcomes. There is an interest in uncovering novel therapeutic options (namely targeted therapies) for this subgroup of patients who often succumb to the disease. Still, the tumor mutational burden of TGCTs is relatively low, so the effect of targeted agents and immunotherapies has been less impressive when compared to other cancers. A better understanding of additional targetable molecular mechanisms is needed in order to effectively treat these patients (5).

It is also important to uncover molecular traits that help to refine current risk stratification of TGCT patients. This will help to improve identification of those patients that will benefit the most from adjuvant chemotherapy, sparing those that can be safely put on active surveillance protocols after surgery from the potential toxicity of systemic treatment. This is important, given the known deleterious side effects of platin-based chemotherapy on such young patients, which will impact their quality of life (6).

In this Research Topic we welcomed contributions on molecular mechanisms of TGCTs (both pre-clinical and clinical), looking for advances in our understanding of this tumor model which can potentially translate into clinical practice.

In this Research Topic, we highlight the work of Ivovic et al., who focused on cisplatin resistance. The authors re-validated their previous results showing that DNA damage levels in peripheral blood mononuclear cells are of prognostic value in GCT patients. In this work, authors add that DNA damage levels are also informative after the first cycle of chemotherapy, serving as prognosticators for progression-free survival. Additionally, a modification of the comet assay is shown to be more reliable than the classical method, increasing reproducibility of the assay for measuring DNA damage.

Cabral et al. explored copy number alterations in *KRAS* gene, showing that this is a frequent event, particularly in non-seminomas. This may be due to the presence of gains of 12p, a marker of most postpubertal-type TGCTs. Despite the overall low mutational burden characteristic of TGCTs, the authors found mutations in 11 of 15 genes assessed with a focused sequencing panel, including *BRAF* and *MET*, which are therapeutically targetable.

Finally, Młynarczyk et al. analyzed the immunoexpression of markers classically related to cell adhesion in a seminoma cohort compared to normal testicular parenchyma, and Favero et al. reported two cases of paraneoplastic hyperthyroidism occurring in non-seminoma patients due to cross-reaction of HCG with the receptor of thyroid stimulating hormone.

To conclude, molecular investigations of TGCTs, when supported by accurate clinical and histopathological annotation, will contribute to generating novel biomarkers and more individualized treatment strategies for these young patients.

## Author contributions

JL: Writing – original draft, Writing – review & editing. AA: Writing – original draft, Writing – review & editing. MM: Writing – original draft, Writing – review & editing.
